# Identification and Characterization of a Novel Calcium-Activated Apyrase from *Cryptosporidium* Parasites and Its Potential Role in Pathogenesis

**DOI:** 10.1371/journal.pone.0031030

**Published:** 2012-02-07

**Authors:** Patricio A. Manque, Ute Woehlbier, Ana M. Lara, Fernando Tenjo, João M. Alves, Gregory A. Buck

**Affiliations:** 1 Department of Microbiology and Immunology, Virginia Commonwealth University, Richmond, Virginia, United States of America; 2 Center for the Study of Biological Complexity, Virginia Commonwealth University, Richmond, Virginia, United States of America; 3 Department of Biology, Virginia Commonwealth University, Richmond, Virginia, United States of America; Institut National de la Santé et de la Recherche Médicale - Institut Cochin, France

## Abstract

Herein, we report the biochemical and functional characterization of a novel Ca^2+^-activated nucleoside diphosphatase (apyrase), CApy, of the intracellular gut pathogen *Cryptosporidium*. The purified recombinant CApy protein displayed activity, substrate specificity and calcium dependency strikingly similar to the previously described human apyrase, SCAN-1 (soluble calcium-activated nucleotidase 1). CApy was found to be expressed in both *Cryptosporidium parvum* oocysts and sporozoites, and displayed a polar localization in the latter, suggesting a possible co-localization with the apical complex of the parasite. *In vitro* binding experiments revealed that CApy interacts with the host cell in a dose-dependent fashion, implying the presence of an interacting partner on the surface of the host cell. Antibodies directed against CApy block *Cryptosporidium parvum* sporozoite invasion of HCT-8 cells, suggesting that CApy may play an active role during the early stages of parasite invasion. Sequence analyses revealed that the *capy* gene shares a high degree of homology with apyrases identified in other organisms, including parasites, insects and humans. Phylogenetic analysis argues that the *capy* gene is most likely an ancestral feature that has been lost from most apicomplexan genomes except *Cryptosporidium*, *Neospora* and *Toxoplasma*.

## Introduction

Cryptosporidiosis, caused by the obligate intracellular protozoan *Cryptosporidium*, is a ubiquitous infectious disease that can cause a persistent and chronic diarrhea [1]. Most *Cryptosporidium* infections in humans are self-limiting, but severe disease may occur in immunodeficient hosts, in particular in AIDS patients [2]. Although *Cryptosporidium hominis* (*C. hominis*) is the most prevalent cause of endemic disease in humans, *Cryptosporidium parvum* (*C. parvum*) also can cause serious outbreaks when humans are exposed to contaminated water supplies or are otherwise in close contact with the non-human carriers of this parasite. Effective vaccines and safe non-toxic anti-cryptosporidial drugs are not yet available. The cellular and molecular mechanisms of infection are poorly understood, mostly due to difficulties in propagation of the parasite in culture.

A *Cryptosporidium* infection is initiated when the host ingests oocysts from which invasive sporozoites emerge and infect enterocytes [3]. Clearly, interactions between macromolecules of the parasites and host cells are of critical importance for the establishment of the infection and consequent survival of the parasite. Thus, pathogenic factors such as parasite proteins or macromolecules responsible for attachment or invasion, or factors that block host cell responses, are ideal targets for drug and vaccine development.

Nucleotide mediated signaling plays a central role in maintaining homeostasis in many tissues. Thus, ecto-nucleotidases are major players in the regulation of purinergic signaling, modulate inflammation and immune responses in Langerhans cells [4], and lead to cardioprotection and protective responses to hypoxia/ischemia in mice [5], [6]. As signaling molecules, extracellular nucleotides also serve as danger signals induced by pathogen infection as well as cell or tissue injury, triggering various cellular events such as proliferation, differentiation and chemotaxis [7]. Recently, high ecto-nucleotidase activity of several protozoan parasites - including *Toxoplasma gondii*, *Entamoeba histolytica*, *Leishmania tropica*, *Leishmania amazonesis*, *Trypanosoma cruzi*, *Trypanosoma brucei*, and *Tritrichomonas foetus* - has been shown to interfere with the extracellular signaling of the host and affect the virulence and pathogenesis of these organisms [8], [9], [10], [11], [12], [13], [14], [15], [16], [17]. Thus, it has been suggested that these enzymes play a role in the pathogenicity of these parasites by controlling the host cell response to infection, specifically by: (i) protecting the parasite from the cytolytic effects of extracellular ATP, (ii) regulating ectokinase substrate concentrations, (iii) preventing activation of signal transduction cascades associated with cellular injury, and (iv) facilitating cellular adhesion [18], [19], [20], [21], [22], [23], [24], [25], [26], [27], [28], reviewed in [28].

Among ecto-nucleosidases, Ecto-ATPases, or E-ATPases, are cell-surface enzymes that hydrolyze a range of extracellular nucleoside triphosphates (NTPs) and nucleoside diphosphates (NDPs). Most of the E-ATPases are apyrases (ATP diphosphohydrolases, EC 3.6.1.5), enzymes that were originally defined as those that catalyze the hydrolysis of both adenosine triphosphate (ATP) and adenosine diphosphate (ADP) to adenosine monophosphate (AMP) and inorganic phosphate (P_i_) [18]. The majority of known apyrases belong, on basis of sequence homology, to the CD39 family. CD39, also known as ENTPD1 (ectonucleoside triphosphate diphosphohydrolase 1), is an integral plasma membrane protein with two transmembrane domains and a large heavily glycosylated extracellular region with nucleoside triphosphate diphosphohydrolase activity [18], [29], [30]. However, a novel and evolutionarily distinct apyrase, that differs from the CD39 family in amino acid sequence as well as its exclusive calcium-dependent functionality, has been identified in the salivary glands of blood-sucking bed bug *Cimex lectularius* [31]. A series of homologs to the *Cimex* gene were recently found in other blood-sucking insects, as well as in vertebrates, including humans, indicating that these enzymes represent an evolutionarily widespread family of proteins [31], [32], [33], [34], [35], [36], [37], [38].

Herein we describe for the first time the biochemical and functional characterization of an apyrase from *C. hominis*, designated CApy, its potential role during the infection, and the resulting implications for pathogenesis during cryptosporidiosis.

## Materials and Methods

### Ethics statement

All mice were housed at the vivarium at Virginia Commonwealth University (VCU) in an AAALAC-accredited facility and experimentation followed VCU Institutional Animal Care and Use Committee approved protocols (VCU IACUC Approved Protocol AM10329, Cryptosporidium Reverse Vaccinology). This study was carried out in strict accordance with the recommendations in the Guide for the Care and Use of Laboratory Animals of the National Institutes of Health (NIH).

### Mammalian cell culture and *C. parvum*

Intestinal epithelial HCT-8 cells were obtained from ATCC (CCL-244) and grown in 75-cm^2^ flasks in Dulbecco's modified Eagle's medium (DMEM, Invitrogen) containing 10% fetal calf serum, 25 mM HEPES, 100 units penicillin, and 100 µg of streptomycin per ml at 37°C in 5% CO_2_ as described [39]. *C. parvum* oocysts were purchased from the University of Arizona. Oocysts were stored at 4°C until use.

### Plasmid construction

The sequence encoding the *C. hominis* apyrase gene (CApy) (Chro. 60194) lacking the N-terminal signal sequence was obtained by PCR amplification from *C. hominis* genomic DNA and cloned into the pTriEx-4 Ek/LIC vector (Novagen) using the following primers: *apyLICF* 5′GACGACGACAAGATGATAGACGAAAGGAGGGTTTG3′, and *apyLICR* 5′GAGGAGAAGCCCGGTTTATATAAATTCTATCCCCTCGTATTTG3′. The 5′ end of the primers incorporated the LIC (ligation independent cloning) sequences (underlined). The amplified CApy product was ligated into pTriEx-4 after treatment with LIC-qualified T4 DNA polymerase as described by the manufacturer (Novagen). *E. coli* strain NovaBlue (Novagen) and *E. coli* strain BL21(DE3) (Novagen) were used for plasmid maintenance and protein expression, respectively. The resulting protein is fused to an N-terminal His_6_- and S-tag with a predicted molecular mass of 41,014 Da, and is referred to as recombinant CApy, designated rCApy.

For production of an unrelated control protein (composed of an N-terminal His_6_-tag, Nus-protein, and C-terminal His_6_- and S-tag), the pET44 Ek/LIC vector (Novagen) transformed into *E. coli* strain BL21(DE3) was used. The resulting protein with a molecular mass of 61,523 Da is herein referred to as Nus.

### Expression and purification of rCApy protein

The *E. coli* strain BL21(D3) transformed with pTriEx-4/CApy was cultured aerobically in TB medium (Overnight Express™ Autoinduction System, Novagen) supplemented with ampicillin (100 µg/ml) at 37°C under constant agitation. The rCApy protein C-terminally fused to a His_6_/S-Tag was expressed in inclusion bodies (not shown). Cell pellets were resuspended in BugBuster protein extraction reagent (Novagen) with Lysonase™ solution (Novagen), and incubated for 30 min at room temperature to induce lysis. After centrifugation at 39000×g (Sorvall SS-34 rotor) for 30 min at 4°C, the supernatant was discarded and the pellet was resuspended in BugBuster protein extraction reagent with rLysozyme™ solution (Novagen). Following incubation for 5 min at room temperature, 6 volumes of 1∶10 diluted BugBuster protein extraction buffer was added and mixed by vortexing for 1 min. The suspension was centrifuged for 15 min at 39000×g (Sorvall SS-34 rotor) at 4°C and the supernatant was discarded. rCApy inclusion bodies were resuspended in 20 mM sodium phosphate, pH 7.4, 500 mM NaCl, 6 M guanidine hydrochloride (buffer 1), and vortexed for 5 min. The buffer was adjusted to 20 mM sodium phosphate pH 7.4, 500 mM NaCl, 4 M guanidine hydrochloride, 10 mM imidazole (buffer 2) and applied to Ni^2+^ chelate affinity chromatography using a column with a 1 ml bed volume (GE Healthcare). The solubilized rCApy inclusion bodies were loaded onto a Ni^2+^- column in buffer 2 (GE Healthcare). Bound proteins were washed with buffer 3 (buffer 1 with 25 mM imidazole) and eluted with buffer 4 (buffer 1 with 500 mM imidazole). The eluted material was renatured by dialysis overnight against a minimum of 50 volumes of 100 mM Tris, pH 8.0, 1 M arginine, 2 mM EDTA, 1 mM GSH (glutathione reduced), 0.1 mM GSSG (glutathione oxidized), 5% glycerol at 4°C. Finally, after additional dialysis overnight at 4°C against a minimum of 200 volumes of PBS (phosphate buffered saline), pH 7.4, or 20 mM MOPS, pH 7.4, the purity of the CApy protein preparation was examined by SDS-PAGE analysis. Protein concentrations were determined according to Bradford (Biorad).

To purify Nus, the supernatant after bacterial cell lysis as described above was diluted 1∶1 with 20 mM sodium phosphate pH 7.4, 500 mM NaCl, 10 mM imidazole (buffer 5) and loaded onto a Ni^2+^- column (GE Healthcare) equilibrated with buffer 5. Column bound proteins were washed with buffer 6 (buffer 5 with 50 mM imidazole) and eluted with buffer 7 (buffer 5 with 500 mM imidazole). The eluted material was dialyzed overnight 4°C against a minimum of 200 volumes of PBS, pH 7.4.

### Production of antibodies against rCApy

Groups of five female 6 to 8 week old C57BL/6 mice (Jackson Laboratory, MA) were immunized i.p. on day 0 with 20 µg purified rCApy or Nus formulated in Freund's complete adjuvant. Mice were boosted i.p. twice on day 21 and 42 with 20 µg purified rCApy or Nus formulated in incomplete Freund's adjuvant. Blood samples were collected on day 63 and sera were prepared, pooled, and stored at −20°C. The serum against Nus protein was used as an unrelated control serum.

### SDS-PAGE and Western blot analysis

To obtain sporozoites, 1×10^8^ *C. parvum* oocysts were washed three times (5,000×g for 4 minutes at 4°C) with Hanks' Balanced Salt Solution, transferred to excystation medium (0.75% Sodium Taurocholate and 0.25% Trypsin in Hanks' medium) and incubated 37°C for one hour.

For preparation of soluble and insoluble fractions of oocysts and sporozoites, parasites were lysed in a Nonidet P-40 based lysis buffer (1% Nonidet P-40, 50 mM Tris/HCl, pH 7.4, 150 mM NaCl, 20% glycerol, proteinase inhibitor cocktail (Roche)) for 20 min at room temperature. After centrifugation at 16000×g and 4°C the supernatant (soluble fraction) was collected and the pellet (insoluble fraction) was dissolved in Nonidet P-40 based lysis buffer.

To remove N and O-linked oligosaccharides from native CApy, sporozoites obtained as described above were treated with the Enzymatic Carbo Release Kit™ (QAbio, San Mateo, CA) as recommended by the manufacturer. In brief, 2×10^8^ sporozoites in 385 µl of water were mixed with 55 µl of Carbo Release buffer. After addition of 27.5 µl denaturation buffer, the sample was boiled for 5 min, chilled on ice, and supplemented with 27.5 µl Triton-X. For incubation with various combinations of carbohydrate removing enzymes, the sporozoite extract was distributed into 1.5 ml microfuge tubes (45 µl each), and 1 µl each of the following enzymes was added: PNGase, sialidase, ß-galactosidase, glucosaminidase and/or O-glycosidase, as recommended by manufacturer. After incubation at 37°C for 16 hr, protein loading buffer was added, the samples were incubated at 100°C for 5 min, and an equivalent of 2.5×10^6^ sporozoites was subjected SDS-PAGE (12% polyacrylamide). Separated proteins were transferred to PVDF nitrocellulose membrane (Millipore) for 90 min at 350 mA and 4°C. Following blocking for 1 h at room temperature with 10 mM Tris, pH 8.0, 150 mM NaCl, 0.05% Tween20 (TBST) containing 2% milk powder (blocking buffer); the nitrocellulose membrane was incubated for 1 hour at room temperature with mouse anti-rCApy serum diluted 1∶500 in blocking buffer. After 3 washes with TBST, the membrane was further incubated with anti-mouse IgG HRP-conjugate (Sigma) diluted 1∶5000 in blocking buffer. The final reaction was revealed by chemiluminescence using ECL Western blotting detection reagent (Pierce) and BioMax light film (Kodak) as recommended by the manufacturers.

### Apyrase activity assays

Apyrase activity assays were conducted in 96-well microtiter plates at 23°C in a final volume of 80 µl of 20 mM MOPS, pH 7.4, 100 mM NaCl containing 0.5 µM rCApy and 2.5 mM NTPs, NDPs, or NMPs. Additionally, CaCl_2_, MgCl_2_ and/or EDTA the solutions to an end concentration of 5 mM. After 10 min, 20 µl of Chan's reagent (SensoLyte™ MG Phosphate Assay Kit, AnaSpec) was added to determine the amount of inorganic phosphate (P_i_) formed. After 5 min of gentle shaking, absorbance was measured at 630 nm. The units used for enzyme activity are µmol of P_i_ generated per mg of protein per hour.

### HCT-8 cell binding assay

Cell binding assays were performed similar as previously described [40]. Briefly, HCT-8 cells (1×10^5^/well) were seeded in 96-well microtiter plates (Costar) and grown overnight at 37°C. After fixation with 4% paraformaldehyde in PBS, pH 7.4, for 1 h at RT, the cells were washed three times with PBS, pH 7.4, blocked for 1 hour at room temperature with PBS, pH 7.4, containing 10% FCS (PBS-FCS), washed three times with PBS, pH 7.4, and incubated with varying concentrations of rCApy or Nus protein as an unrelated protein control, in PBS-FCS. After 1 h at 37°C, the cells were washed four times with PBS, pH 7.4, containing 0.05% Tween20 (PBS-Tween), and incubated for 1 h at 37°C with mouse anti-His_6_-HRP conjugated antibody 1∶10,000 (SantaCruz) diluted in PBS-FCS. After four washes, PBS-Tween substrate buffer containing *o*-phenylenediamine (SIGMA*FAST* OPD™) was added, the cells were incubated for 30 min and the OD was measured at 450 nm.

### Cell invasion assay

Sporozoites (1×10^6^) were incubated with 1∶10 diluted mouse anti-rCApy serum or non-related control serum (anti-Nus) for 30 min at 37°C, washed three times with PBS, pH 7.4, and added to 24-well plates containing a monolayer of HCT-8 cells in a ratio of 4 parasite:1 cell. Following incubation under culture conditions for 3 hours, extracellular parasites were removed by washing three times with PBS, pH 7.4, and the plates were returned to the incubater. Cells were collected 24 hours post-infection RNA was extracted from infected cells using the RNAqueous system (Ambion) following the manufacturer's recommendations. RNA samples were stored at −80°C until quantification of *C. parvum* rRNA by RT-PCR.

### Real-time quantitative RT-PCR

RT-PCR was used to quantify the *in vitro* infection rate of HCT-8 cells by *C. parvum*. Thus, RNA samples from HCT-8 cells infected with *C. parvum* sporozoites pretreated with mouse anti-rCApy serum and the corresponding control (mouse anti-Nus serum) were incubated with TURBO DNA-free DNase (Ambion) following the manufacture's instructions and used for Real-Time RT-PCR analysis using TaqMan™ technology. Primers and probes specific for *Cryptosporidium* rRNA and human rRNA were designed using Primer Express® version 2.0 (ABI). For each target, forward and reverse primers and an internal probe were synthesized. Probes were synthesized with 5′ end linked FAM (6-carboxyfluoresceine) and 3′ end fluorescent TAMRA (6-carboxytetramethylrhodamine) dyes. Amplification and analysis were performed in our ABI7900HT instrument. The infection rate was obtained by calculating the ratio of human rRNA versus *Cryptosporidium* rRNA, with 100% infection represented by the negative control (treated with anti-Nus serum).

### Sequence analysis

Putative signal peptide cleavage sites and asparagine-linked glycosylation sites of CApy were determined using SignalP 3.0 (http://www.cbs.dtu.dk/services/SignalP/) and NetNGlyc 1.0 (http://www.cbs.dtu.dk/services/NetNGlyc/). The theoretical molecular weights and isoelectric points were determined with PeptideMass (http://www.expasy.ch/tools/peptide-mass.html).

### Phylogenetic analysis of CApy

Putative orthologs of CApy were identified by BLAST [41] search of the CApy protein sequence against NCBI GenBank non-redundant protein database (nr), with a threshold of 1E-6 for the expectancy value E. All the identified protein sequences were aligned with ClustalX [42] and manually checked for adequate aligned length (>75% of the protein aligning). To expedite subsequent bootstrap analyses and simplify the final tree, phylogenetic analysis was performed by maximum likelihood using PHYML [43] in two steps: first, a preliminary analysis (without bootstrap and using less thorough tree space search strategies) identified possible in-paralogs as well as out-paralogs that reflected the same phylogenetic pattern. These sequences were then removed from the alignment, and the sequences were realigned and reanalyzed to check whether the phylogeny remained stable after removal of the paralogs. The second analysis, involving only sequences remaining after the first step, was performed with one hundred bootstrap replicates and the SPR strategy [44] for the search of the optimal trees. The amino acid substitution model used was LG with 4 categories of gamma-distributed substitution rates, as selected by ModelGenerator [45]. Table S1 lists the GenBank accession numbers for the 109 sequences utilized in phylogenetic reconstruction.

## Results

### Identification and sequence analysis of a *C. hominis* apyrase (CApy)

**Figure 1:**
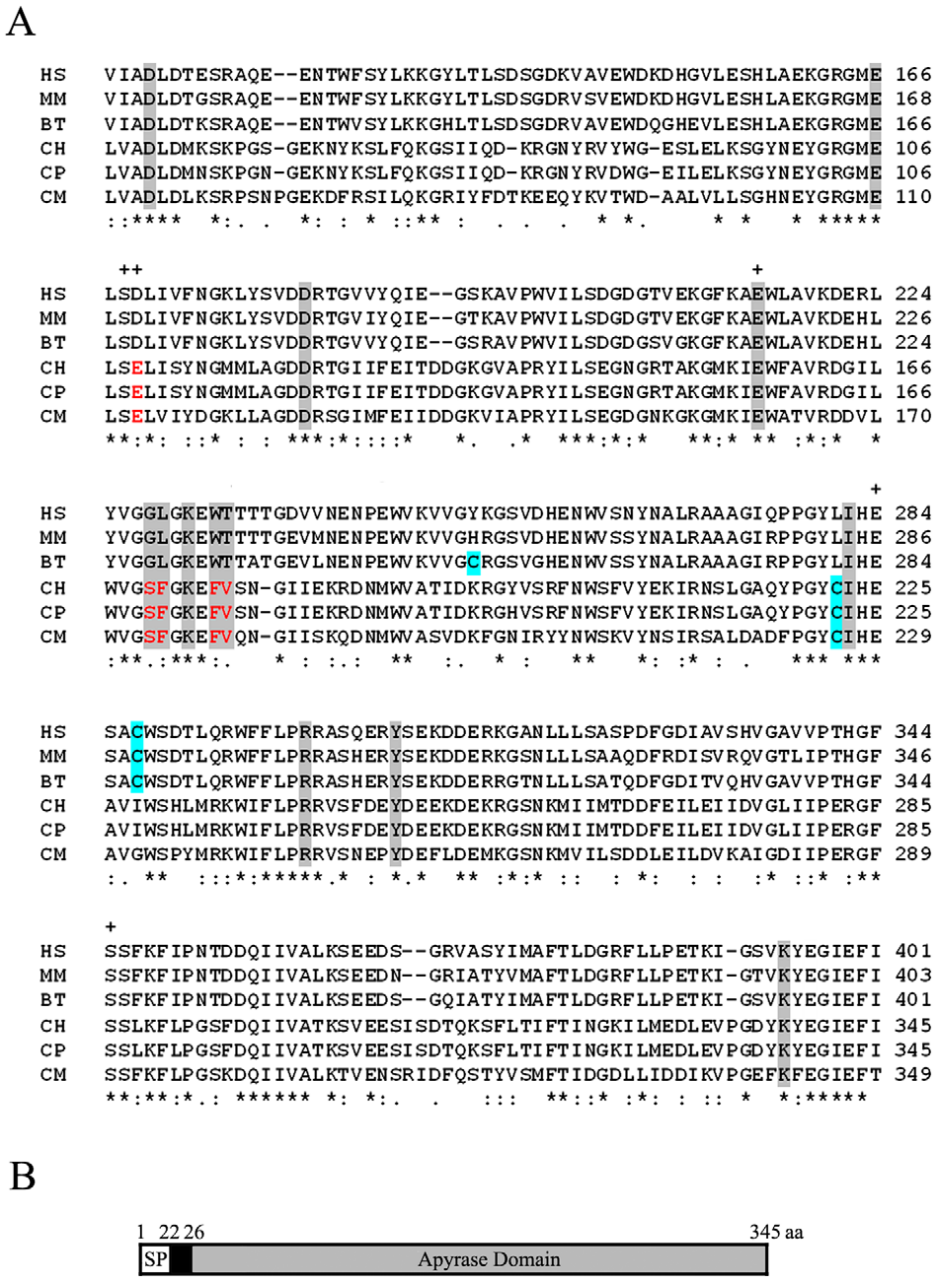
Schematic outline of CApy and multiple sequence alignment of the apyrase domain of Cryptosporidium sp. with apyrase homologs of their respective hosts. A. Alignment of selected apyrase sequences. Sequence alignment was performed using the CLUSTAL 2W algorithm. The GenBank accession numbers of sequences are given in parentheses. CH, *Cryptosporidium hominis* (XP_666945); CP, *Cryptosporidium parvum* (XP_627524); CM, *Cryptosporidium muris* (XP_002140694); HS, *Homo sapiens* (NP_620148); MM, *Mus musculus* (EDL34666), BT, *Bos Taurus* (XP_596269). The enzymatic activity of the human apyrase has been established by biochemical analysis [56], therefore residues important for nucleotide and Ca^2+^ binding in the human apyrase and the predicted counterparts in corresponding apyrases of other species are indicated by gray shadows and pluses. Residues that differ from the human sequence at positions with potential impact on enzymatic activity are colored red. Cysteine residues are shadowed light blue. B. CApy is a protein comprising 345 aa, including a 22-aa signal peptide (SP), and an apyrase domain spanning residues 26–345.

Analysis of the recently sequenced *C. hominis* and *C. parvum* genomes identified a single ecto-ATP diphoshohydrolase (apyrase, EC 3.6.1.5) in each sequence that displays high similarity to the human soluble calcium-activated nucleotidase 1 (SCAN-1, Fig. 1A) (GenBank accession numbers EAL36710, and EAK89829). The *Cryptosporidium* apyrase (CApy) is a single copy gene with a predicted open reading frame of 1035 bp coding for a 345 amino acid protein. Further characterization of CApy amino acid protein sequence predicted a signal peptide with a cleavage site between residues 22 and 23. The cleavage results in a protein of 323 amino acids, with a predicted molecular mass of 36,932 Da and an isoelectric point of 5.23 (Fig. 1B). No GPI anchor or other known cell targeting signals were found, suggesting that the protein is either attached to the membrane by a hydrophobic stretch or secreted. In addition, N-linked glycosylation sites were not detected.

### Expression, Purification and Refolding of recombinant CApy

**Figure 2:**
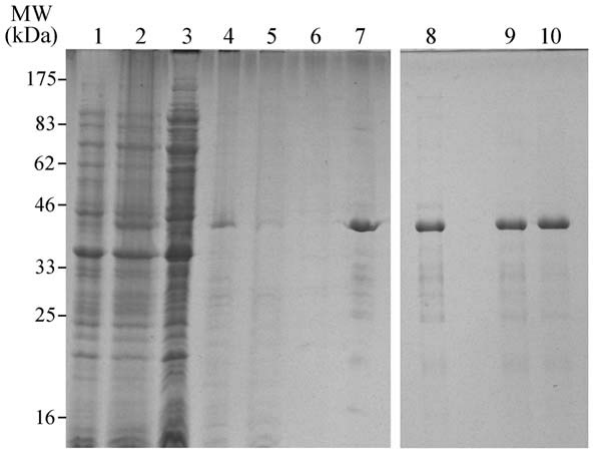
Expression, Purification and Refolding of recombinant CApy. The electrophoretic analysis (SDS-PAGE, 12% PAA under reducing conditions) shows extracts of non-induced and induced *E. coli* cultures (lane 1 and 2) bearing the CApy gene in the pTriEx-4 expression vector from samples taken at different steps of protein purification (lanes 3–10). Following bacterial cell lysis the soluble (supernatant, lane 3) and insoluble (pellet, lane 4) fractions show that CApy was mostly found in the insoluble fraction in form of inclusion bodies (IBs). The solubilized IBs (see Materials and Methods) were loaded onto a Ni^2+^ chelate column and purified under denaturing conditions, flow-through (lane 5), wash (lane 6), and elution (lane 7) fractions were collected. The eluate containing the purified CApy was refolded by dialysis against folding buffer (lane 8), which was subsequently dialysed against PBS, pH 7.4 (lane 9) or 20 mM MOPS, pH 7.4 (lane10).

The *C. parvum* and *C. hominis* apyrase amino acid sequences are 98% identical. Herein, we describe the cloning, expression and purification of recombinant CApy derived from *C. hominis*, designated rCApy. Thus, the DNA sequence encoding amino acid residues 34–345 excluding the signal peptide of the gene was amplified from *C. hominis* genomic DNA. The resulting 936-bp fragment was inserted into the pTriEx-4 vector (Novagen) C-terminally fused to a His_6_-S-tag. As shown in Fig. 2, rCApy was expressed in *E. coli* in inclusion bodies (IBs) and was therefore solubilized as described in the Materials and Methods. Subsequent purification by Ni^2+^- chelate chromatography under denaturing conditions led to a highly purified and homogenous product (Fig. 2, lane 7). During refolding of rCApy, little or no protein was lost due to precipitation (Fig. 2, lane 8). Nevertheless, in subsequent dialysis against PBS, pH 7.4, or MOPS pH 7.4 (Fig. 2, lanes 9 and 10), a significant fraction of the refolded protein precipitated when protein concentrations exceeded approximately 0.35 mg/ml. A similar dependency on protein concentration for successful refolding was reported for human SCAN-1 [38].

### Expression and localization of CApy in infective forms of *C. parvum*

**Figure 3:**
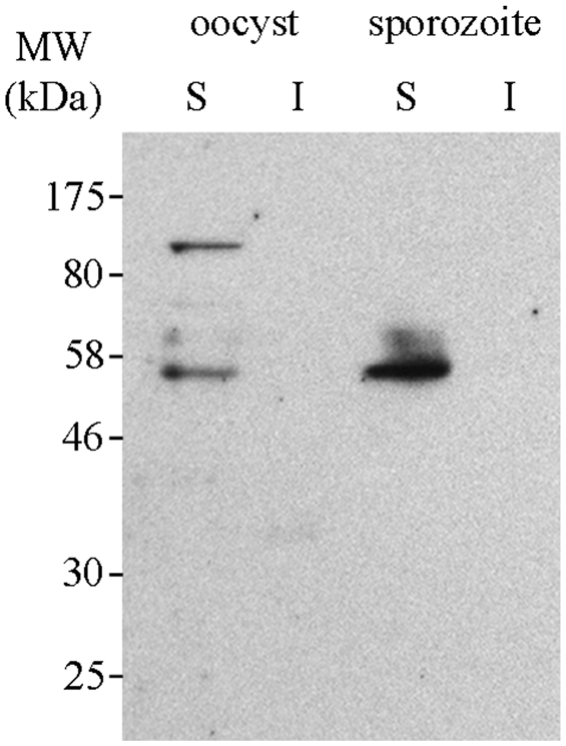
Expression of CApy in oocysts and sporozoites. CApy is expressed in oocysts and sporozoites. Aliquots of NP-40 soluble (S) or insoluble (I) *C. parvum* oocyst and sporozoite fractions were resolved by 12% SDS-PAGE, transferred to nitrocellulose and probed with mouse anti-rCApy serum as described in the Materials and Methods.

To determine the expression and localization of the CApy protein in *Cryptosporidium* parasites, we performed Western blot experiments. Since *C. hominis* is very difficult to obtain, we used *C. parvum* in this and the following experiments where parasites are required. Since the genomes of these two parasites exhibit ∼95% sequence identity and the amino acid sequences of the CApy proteins are nearly 98% identical [46], [47], we do not expect major differences in results obtained using *C. parvum* parasites. Western blot analysis using polyclonal antibodies against rCApy showed that the native protein is in the detergent-soluble fraction of oocysts and sporozoites with an apparent molecular mass of ∼50 kDa (Fig. 3). The apparent ∼50 kDa molecular mass suggests that the protein may be subject to post-translational modifications. Higher molecular mass bands were also observed, particularly in the oocyst fraction (Fig. 3), suggesting that CApy could form multimer complexes. Previously we showed using polyclonal mouse antibodies raised against rCApy that this parasite protein is membrane associated with polar localization in the infected sporozoites [48]. The intriguing possibility that CApy is associated with and secreted by the apical complex in sporozoites remains to be further explored.

**Figure 4:**
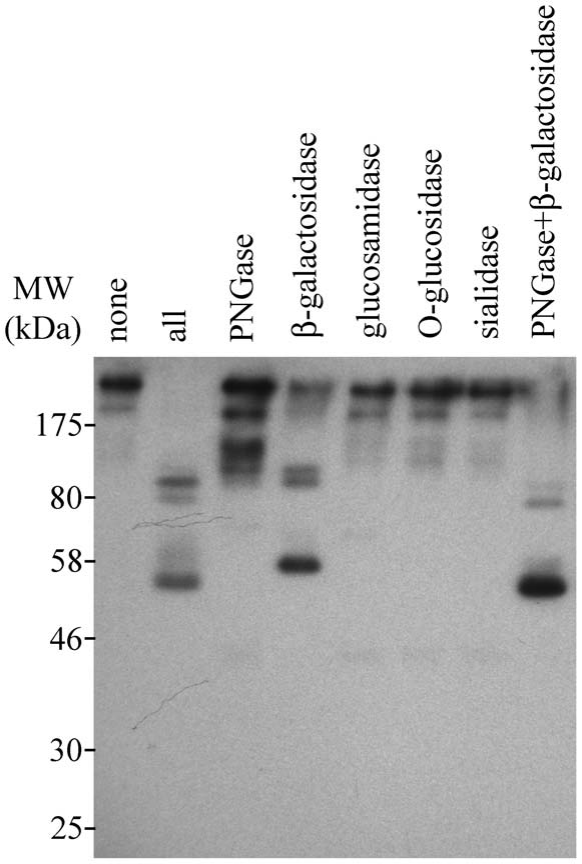
Glycosylation of CApy in sporozoites. *C. parvum* sporozoites were treated as recommended in the Enzymatic Carbo Release Kit™ (QAbio, San Mateo, CA) for identification of glycosylation. In brief, sporozoites were suspended in Carbo Release buffer, and denaturation buffer was added. After incubation at 100°C for 5 min, samples were chilled on ice and Triton-X was added. The enzymes PNGase, Sialidase, ß-Galactosidase, Glucosaminidase, O-Glycosidase were added alone or in combinations. Following incubation at 37°C for 16 hours, protein loading buffer was added and samples were incubated again at 100°C for 5 min. Proteins were separated by 12% SDS-PAGE, transferred to nitrocellulose and probed with mouse anti-rCApy serum.

To investigate the possible presence of N- and O- linked oligosaccharides on CApy, we performed enzymatic deglycosylation experiments on the proteins in sporozoite extracts and analyzed the resulting proteins by Western blot analysis using CApy-specific antibodies (Fig. 4). Treatment with glucosamidase, O-glucosidase, or sialidase showed little effect on the migration of CApy. In contrast, treatment with PNGase or ß-galactosidase revealed a major shift in the molecular mass of CApy, with ß-galactosidase having the most pronounced effect. Nevertheless, even after deglycosylation with both PNGase and ß-galactosidase the most prominent band detected in sporozoites was significantly higher, ∼50 kDa, than the expected molecular weight for CApy, ∼39 kDa. Moreover, reduction of samples in the presence 100 mM DTT (1,4-dithio-DL-threitol) before separation in SDS-PAGE did not change the outcome of the Western blot (data not shown). These results suggest that CApy in its mature form is posttranslationally glycosylated in an N-linked fashion. Additional posttranslational modifications may also be present since a larger than the expected protein species was obtained after deglycosylation.

### Activity of CApy requires Ca^2+^ and is highest against UDP and GDP

**Figure 5:**
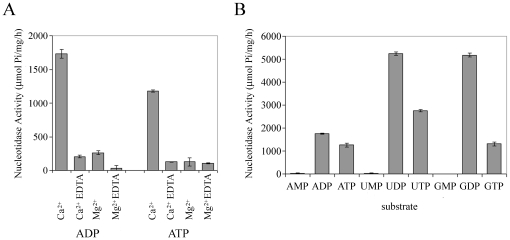
Enzymatic characterization of recombinant CApy. Apyrase activity was measured in 20 mM MOPS buffer, pH 7.4, and a final nucleotide concentration of 2.5 mM. Activity units are micromoles of P_i_ liberated per milligram of protein per hour at 23°C. The graphs present the mean and standard deviations of triplicate experiments. **A**. Cofactor specificity of rCApy. ATPase and ADPase activity of rCApy was measured in presence of either 5 mM CaCl_2_ or 5 mM MgCl_2_ with or without addition of 5 mM EDTA. **B**. Substrate preference of rCApy. Assays were done in presence of 5 mM CaCl_2_.

We evaluated the nucleotidase activity of rCApy using a variety of substrates and divalent cations. ATPase and ADPase activities were measured at 2.5 mM total nucleotide concentrations in the absence of divalent cations, as well as in presence of 5 mM CaCl_2_, or MgCl_2_ (Fig. 5A). Nucleotidase activity was only detected in presence of calcium ions. To determine enzyme substrate specificity of CApy, a variety of nucleoside mono-, di-, and triphosphates were used as substrates (2.5 mM final nucleotide concentrations) in the presence of 5 mM CaCl_2_ (Fig. 5B). The activity for the preferred substrates UDP and GDP is high, whereas activities towards ATP and GTP are lowest. No measurable hydrolysis of any investigated monophosphate was detected. These results reflect those expected for human apyrase SCAN-1.

### Recombinant CApy binds to the HCT-8 cells in a dose dependent fashion

**Figure 6:**
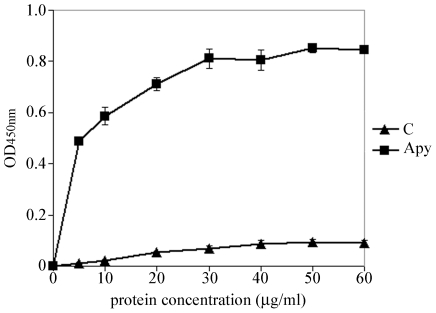
Binding of recombinant CApy to intestinal epithelial cells. Increasing concentrations of protein in the range of 5–60 µg/ml, rCApy or control protein, were added to 96-well plates containing formaldehyde-fixed adherent HCT-8 cells (as described in Material and Methods. Interactions of proteins, both carrying a His_6_-tag, with the HCT-8 cell surface were detected by ELISA using anti-His_6_ HRP-conjugated antibody. The bound antibody was revealed by using o-phenylenediamine as a substrate. Respective results of one of three experiments are shown, and are expressed as absorbance at 450 nm. The values are the means of one experiment performed in triplicates, error bars represent standard deviations. An unrelated bacterial protein (Nus) was used as a control (C).

To assess the possibility that CApy may play a role in the attachment of *Cryptosporidium* sporozoites to enterocytes, we measured the ability of recombinant CApy to bind HCT-8 cells. Binding assays were performed by adding various concentrations of rCApy to paraformaldehyde fixed HCT-8 cells as described in the Materials and Methods. As shown in Fig. 6, rCApy binds to HCT-8 cells in a dose-dependent and saturable manner. No binding was observed when an unrelated protein (bacterial Nus) was used in identical assays.

### Antibodies directed against rCApy block invasion of HCT-8 cells by *C. parvum*

**Figure 7:**
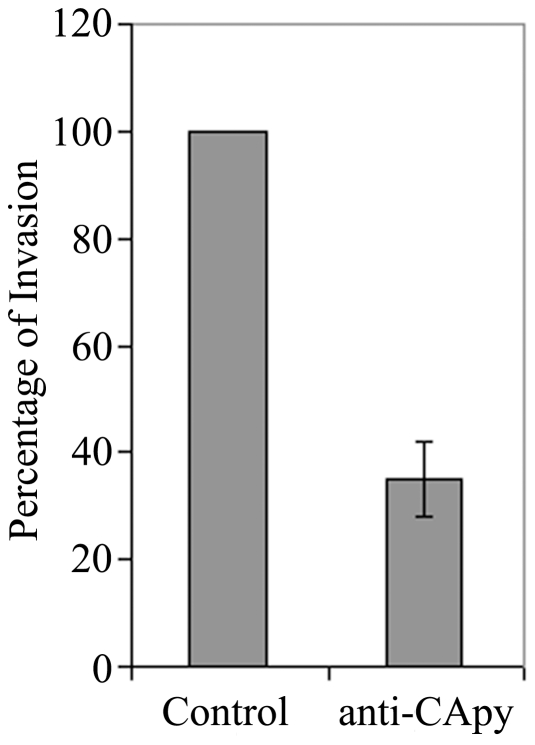
CApy-specific serum blocks C. parvum infection of HCT-8 cells. *C. parvum* sporozoites were preincubated with mouse anti-rCApy serum or unrelated control serum (dilution 1∶10), followed by incubation with HCT-8 cells for 24 hours and infection was quantified by RT-PCR as described in the Materials and Methods. The values are the means three independently performed experiments performed, error bars represent standard deviations.

To further gain insight into the role of CApy during parasite invasion, we performed *in vitro* invasion assays using HCT-8 cells. Thus, sporozoites were pre-incubated with mouse anti-rCApy serum or unrelated control serum and the infection rate was measured using quantitative RT-PCR (Fig. 7). In these experiments, antibodies against rCApy were able to reduce the invasion of enterocytes by sporozoites by over 60%, suggesting that CApy plays a role during the early stages of infection.

### Sequence and phylogenetic analysis of apyrase sequences

**Figure 8:**
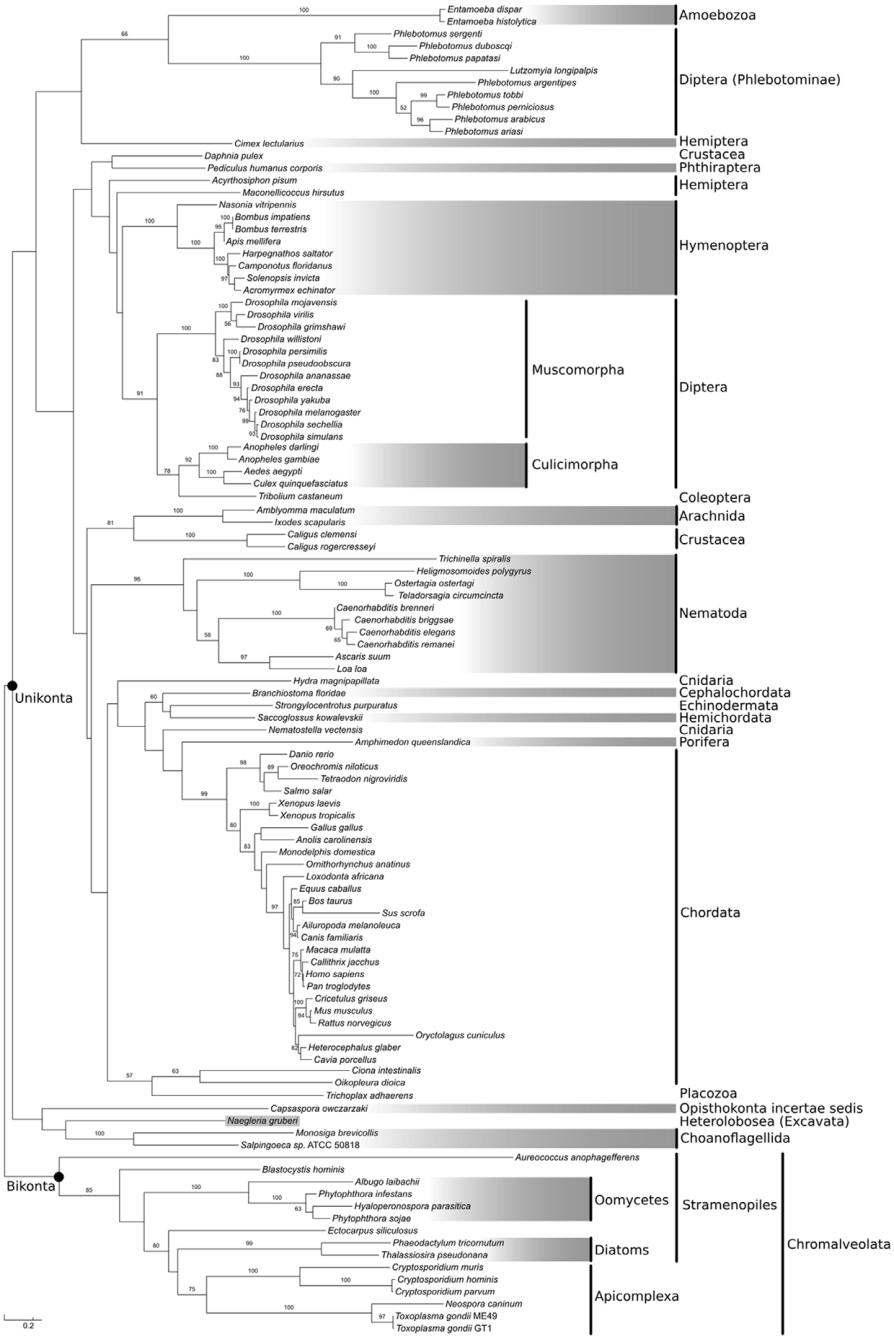
Maximum likelihood phylogenetic tree of all identified CApy orthologs. Numbers at the nodes represent bootstrap support (only numbers above 50 are shown).

To perform a reconstruction of the evolutionary history of CApy, we identified putative orthologs in as many genomes as possible (Table S1) and used these sequences to perform a phylogenetic analysis (Fig. 8, and see Materials and Methods). The analysis showed that among the Unikonta [49], [50], a CApy ortholog is present in Choanozoa, the Amoebozoa, in one Opisthokonta of uncertain phylogenetic affiliation (*Capsaspora*), and in several Metazoa, but not in any fungi. Among the Bikonta, CApy orthologs only seem to be present in the Chromalveolata, comprised of Alveolata (which includes apicomplexan parasites) and Stramenopiles, and in one Excavata, *Naegleria gruberi*, which grouped at a deep divergence, and with low bootstrap support, with the Unikonta. The grouping of Apicomplexa and Stramenopiles has strong bootstrap support, as have the internal groupings of cryptosporidia, diatoms and oomycetes. The branch connecting *Toxoplasma* and *Neospora* with *Cryptosporidium* is relatively well supported, with a bootstrap value of 75 (only values above 50 are shown on Fig. 8). Other known Bikonta (Excavata, Plantae and Rhizaria) genomes seem to lack an ortholog of this protein, with the exception of the excavate *Naegleria*, as noted above.

Multiple sequence alignment of these orthologs from protozoan, invertebrate, and vertebrate organisms shows that most variations occur in the N-terminus, possibly reflecting differences in the secretory systems, as well as possible different localizations of the protein, depending on the specific function in the respective organism. Nevertheless the apyrase domain, and in particular the structurally important regions, seem to be highly conserved (data not shown). Surprisingly, orthologs occur in various infectious disease causing eukaryotic parasites, as well as their respective hosts, yet they are apparently absent in yeast and bacteria. Overall, our analysis argues that CApy was present in the progenitors of *Cryptosporidium*, but was lost in other apicomplexans except *Toxoplasma* and *Neospora*, or, less likely, that the gene was picked up by the parasites from more distantly related organisms by lateral gene transfer.

## Discussion

In this study, we have identified and characterized a *C. hominis* gene, CApy, which encodes an ecto-apyrase homolog of a group of calcium-dependent apyrases that catalyze hydrolysis of both nucleoside triphosphate (NTP) and nucleosine diphosphate (NDP) to nucleoside monophosphate (NMP) and inorganic phosphate (P_i_). These enzymes are highly conserved among the Unikonta, including arthropods and vertebrates [31], [32], [33], [34], [36], [37], but much less prevalent in the Bikonta, which includes the apicomplexans.

Initial analysis of the CApy sequence revealed that the protein displays a classical signal peptide sequence, suggesting that the protein is either located on the surface of the parasite or secreted. No GPI anchor or other transmembrane signal was identified. Interestingly, immunofluorescence experiments suggested that the protein is mainly located in the apical region of the parasite, a highly specialized structure containing several organelles known to secret a series of proteins necessary for invasion of the host [3]. Although bioinformatic analysis did not strongly suggest the presence of glycosylation sites, our observations suggest that CApy is heavily glycosylated. It remains to be investigated to what extent glycosylation influences the function of the molecule.

Our recombinant CApy functions as a soluble apyrase with exclusive calcium-dependent activity, consistent with its human homolog ortholog SCAN-1 [34], [38], as well as various insect apyrases from *Cimex lectularius* [31], *Phlebotomus papatasi* [33], and *Lutzomyia longipalpis* [36]. As observed for SCAN-1, UDP and GDP, rather than ADP or ATP, are the preferred substrates of CApy [34], [38].

Several indications suggest that CApy could be involved in the invasion of human enterocytes by *Cryptosporidium*. Thus, CApy seems to be found on the apical surface of sporozoites, binds to the host cell in a dose-dependent manner suggesting the presence of a potential receptor on the host cell surface, and elicits antibodies that significantly block invasion. Our observations support previous reports that indicate that ecto-apyrases play various roles during host pathogen interaction [51]. Recently it was reported, that antibodies directed against an ecto-NTPDase from *Trypanosoma cruzi* and *Leishmania amazonesis* are able to significantly reduce the rate of infection of these parasites [10], [52]. Furthermore chemical inhibition of the ecto-NTPDase led to a reduction of the virulence of *T. cruzi* in *in vivo* experiments [12]. Moreover, Bissagio et al. 2003 [10] suggested that Mg^2+^-dependent ecto-ATPase activity on the surface of *T. cruzi* could stimulate the adherence of the parasite to the host cell and protect from neutrophil attack.

The ability of CApy to inactivate extracellular nucleotides, which generally serve as danger signals that are rapidly produced during the onset of immune responses, suggests other possible roles of CApy during parasite pathogenesis. Moderate to severe infection with *Cryptosporidium* is characterized by mucosal inflammation with neutrophils and macrophages in the lamina propria underlying intestinal epithelial cells, as well as the presence of intraepithelial neutrophils ([53], reviewed in [54]). Cell and tissue damage, hypoxia, leukocyte activation, decreased pH and other stress factors previously described to be caused by this pathogen [7], [55] may lead to the release of large amounts of extracellular nucleotides into the intestinal lumen at the site of inflammation. Our *in vitro* data indicate that CApy may interfere with the interaction of ADP, ATP, UDP, and UTP with different subsets of purinergic receptors, thereby possibly impacting various signaling pathways normally activated during inflammation resulting in modulation of cellular immune responses. In addition to its potential role in attachment, an apyrase expressed on the surface or secreted by the parasite may be an effective counter measure to the release of extracellular nucleotides rapidly secreted at local sites of infection which would otherwise display potent innate immune-enhancing activities undermining successful parasite propagation. Further studies are necessary to test this hypothesis.

Apyrases are broadly distributed among the tree of life and are present in many pathogenic parasites [28]. To gain insight into the evolution of this pathogenic factor we performed phylogenetic analysis of CApy and all orthologs that we could identify from available sequences. Our analysis, using recent multi-gene phylogenies of eukaryotes as a benchmark [49], suggests that CApy was present in the apicomplexan progenitor, but the gene was lost from apicomplexans other than *Cryptosporidium*, *Neospora*, and *Toxoplasma*. Moreover, the phylogenetic analysis indicates that all Chromalveolata sequences form a monophyletic group, suggesting that CApy might have been an ancestral feature of the group that was later lost in other Apicomplexa (e.g., *Plasmodium*, *Theileria*) and the Ciliata (e.g. *Tetrahymena* and *Paramecium*). While *Toxoplasma*, *Neospora* and *Cryptosporidium* branch together, they do so with moderate bootstrap support of 75. This is likely due to the greater sequence divergence of the *Toxoplasma* and *Neospora* apyrases, which can lead to lower phylogenetic signal and more homoplasy with other sequences. Assuming that the phylogenetic groups Bikonta and Unikonta are natural, the absence of this apyrase in other bikonts (all Excavata but *Naegleria*, all Plantae, and possibly Rhizaria, for which no complete genomes were available at analysis time) suggests that either the CApy gene is ancient but was lost in these bikont lineages, or that this gene is novel and was transferred from unikonts to Chromalveolata (or vice-versa) early in evolution of these groups. Our current taxonomic sampling of this gene does not allow a definitive conclusion, emphasizing the need for further genomic studies of more diverse taxa among the unikonts, Chromalveolata, Rhizaria, as well as basal Plantae, e.g. rhodophytes and glaucophytes.

In summary, herein we described a new potential pathogenic factor, the CApy apyrase, in *Cryptosporidium*. CApy may play multiple roles during the infection, including an active participation in the attachment and invasion of *Cryptosporidium* to the host cells. In addition, we provide indirect evidence suggesting that CApy could potentially interfere with extracellular nucleotide signaling responsible for triggering an inflammatory response in injured tissues, possibly delaying it and providing an opportunity for the parasite to successfully establish the infection. Phylogenetic evidence suggests that either this gene was acquired very early in evolution but lost in many of the nearest relatives of *Cryptosporidium*, explaining its otherwise broad distribution among the tree of life and among pathogenic parasites, or, less likely, that *Cryptosporidium*, *Toxoplasma*, and *Neospora* may have acquired the gene by lateral gene transfer. Finally the evidence provided by this study indicates that CApy might be a potential drug target and/or vaccine candidate against cryptosporidiosis.

## Supporting Information

Table S1 Database accession number of sequences used in phylogenetic reconstruction.
(DOC)
