# Supplementary material for: Identification and Characterization of a Novel Calcium-Activated Apyrase from Cryptosporidium Parasites and Its Potential Role in Pathogenesis
Source: PLoS One. 2012 Feb 7;7(2):e31030. doi: 10.1371/journal.pone.0031030 (PMC3280346; doi:10.1371/journal.pone.0031030)
Supplement: Table S1 — Database accession number of sequences used in phylogenetic reconstruction. (DOC) [file pone.0031030.s001.doc]

Supplementary Table S1: Database accession number of sequences used in phylogenetic reconstruction.

| **Organism** | **Accession #** | **Organism** | **Accession #** |
| --- | --- | --- | --- |
| *Cryptosporidium hominis* TU502 | XP_666945 | *Harpegnathos saltator* | EFN89143 |
| *Cryptosporidium parvum* Iowa II | XP_627524 | *Heligmosomoides polygyrus* | AEP27182 |
| *Cryptosporidium muris* RN66 | XP_002140694 | *Heterocephalus glaber* | EHB01018 |
| *Acromyrmex echinatior* | EGI61010 | *Homo sapiens* | NP_620148 |
| *Acyrthosiphon pisum* | XP_001949871 | *Hyaloperonospora parasitica* | NA ***2** |
| *Aedes aegypti* | XP_001652143 | *Hydra magnipapillata* | XP_002160275 |
| *Ailuropoda melanoleuca* | XP_002918427 | *Ixodes scapularis* | EEC06133 |
| *Albugo laibachii* | CCA23536 | *Loa loa* | XP_003136892 |
| *Amblyomma maculatum* | AEO36734 | *Loxodonta africana* | XP_003417275 |
| *Amphimedon queenslandica* | XP_003385658 | *Lutzomyia longipalpis* | AAD33513 |
| *Anolis carolinensis* | XP_003217170 | *Macaca mulatta* | XP_001109105 |
| *Anopheles darlingi* | EFR20412 | *Maconellicoccus hirsutus* | ABN12001 |
| *Anopheles gambiae* | XP_321938 | *Monodelphis domestica* | XP_001371078 |
| *Apis mellifera* | XP_623385 | *Monosiga brevicollis* | XP_001749289 |
| *Ascaris suum* | ADY48370 | *Mus musculus* | EDL34666 |
| *Aureococcus anophagefferens* | EGB04881.1 | *Naegleria gruberi* | XP_002678622 |
| *Blastocystis hominis* | CBK24990 | *Nasonia vitripennis* | XP_001604339 |
| *Bombus impatiens* | XP_003485037 | *Nematostella vectensis* | XP_001623376 |
| *Bombus terrestris* | XP_003398837 | *Neospora caninum* | CBZ55061 |
| *Bos taurus* | XP_596269 | *Oikopleura dioica* | CBY13327 |
| *Branchiostoma floridae* | XP_002206701 | *Oreochromis niloticus* | XP_003450175 |
| *Caenorhabditis brenneri* | EGT33210 | *Ornithorhynchus anatinus* | XP_001517798 |
| *Caenorhabditis briggsae* | XP_001677103 | *Oryctolagus cuniculus* | XP_002723655 |
| *Caenorhabditis elegans* | NP_509283 | *Ostertagia ostertagi* | ADG63133 |
| *Caenorhabditis remanei* | XP_003118023 | *Pan troglodytes* | XP_523734 |
| *Caligus clemensi* | ACO15411 | *Pediculus humanus corporis* | EEB16399 |
| *Caligus rogercresseyi* | ACO11448 | *Phaeodactylum tricornutum* | XP_002181909 |
| *Callithrix jacchus* | XP_002748854 | *Phlebotomus arabicus* | ACS93495 |
| *Camponotus floridanus* | EFN60371 | *Phlebotomus argentipes* | ABA12135 |
| *Canis familiaris* | XP_848726 | *Phlebotomus ariasi* | AAX56357 |
| *Capsaspora owczarzaki* | EFW39988 | *Phlebotomus duboscqi* | ABI20147 |
| *Cavia porcellus* | XP_003464829 | *Phlebotomus papatasi* | AAG17637 |
| *Cimex lectularius* | O96559 | *Phlebotomus perniciosus* | ABB00907 |
| *Ciona intestinalis* | XP_002129596 | *Phlebotomus sergenti* | ADJ54110 |
| *Cricetulus griseus* | XP_003498864 | *Phlebotomus tobbi* | ADJ54077 |
| *Culex quinquefasciatus* | XP_001849098 | *Phytophthora infestans* | PITG_20792 ***3** |
| *Danio rerio* | NP_999908 | *Phytophthora sojae* | 156688 ***** |
| *Daphnia pulex* | EFX76421 | *Rattus norvegicus* | NP_653355 |
| *Drosophila ananassae* | XP_001954201 | *Saccoglossus kowalevskii* | XP_002741956 |
| *Drosophila erecta* | XP_001980563 | *Salmo salar* | NP_001133981 |
| *Drosophila grimshawi* | XP_001994579 | *Salpingoeca sp.* ATCC 50818 | EGD77526 |
| *Drosophila melanogaster* | CAL26011 | *Solenopsis invicta* | EFZ19843 |
| *Drosophila mojavensis* | XP_002000549 | *Strongylocentrotus purpuratus* | XP_792609 |
| *Drosophila persimilis* | XP_002019374 | *Sus scrofa* | XP_003361304 |
| *Drosophila pseudoobscura* | XP_001359657 | *Teladorsagia circumcincta* | CBW38507 |
| *Drosophila sechellia* | XP_002031621 | *Tetraodon nigroviridis* | CAG00216 |
| *Drosophila simulans* | XP_002103865 | *Thalassiosira pseudonana* | XP_002286296 |
| *Drosophila virilis* | XP_002056726 | *Toxoplasma gondii* GT1 | EEE25629 |
| *Drosophila willistoni* | XP_002069949 | *Toxoplasma gondii* ME49 | EEA97186 |
| *Drosophila yakuba* | XP_002097211 | *Tribolium castaneum* | XP_973892 |
| *Ectocarpus siliculosus* | CBJ27122 | *Trichinella spiralis* | XP_003375638 |
| *Entamoeba dispar* | XP_001739797 | *Trichoplax adhaerens* | XP_002110122 |
| *Entamoeba histolytica* | XP_654244 | *Xenopus laevis* | NP_001079373 |
| *Equus caballus* | XP_001490915 | *Xenopus tropicalis* | NP_988940 |
| *Gallus gallus* | NP_001026752 |  |  |
| Note: all accession numbers accessible thorough GenBank, except where noted. NA: not available.  ***** - *Phytophthora sojae* genome project site (http://genome.jgi-psf.org/Physo1_1/Physo1_1.home.html)  ***2** - *Hyaloperonospora parasitica* genome project site, retrieved by searching against genomic DNA sequence (http://genome.wustl.edu/genomes/view/hyaloperonospora_parasitica/)  ***3** - *Phytophthora infestans* genome project site (http://www.broad.mit.edu/annotation/genome/phytophthora_infestans/) | | | |
